# HPLC-MS/MS method applied to an untargeted metabolomics approach for the diagnosis of “olive quick decline syndrome”

**DOI:** 10.1007/s00216-021-03279-7

**Published:** 2021-03-25

**Authors:** Sabrina Di Masi, Giuseppe E. De Benedetto, Cosimino Malitesta, Maria Saponari, Cinzia Citti, Giuseppe Cannazza, Giuseppe Ciccarella

**Affiliations:** 1 Laboratorio di Chimica Analitica, Dipartimento di Scienze e Tecnologie Biologiche ed Ambientali, Università del Salento, Via Monteroni, 73100 Lecce, Italy; 2 Laboratorio di Spettrometria di Massa Analitica e Isotopica, Dipartimento di Beni Culturali, Università del Salento, Via Monteroni, 73100 Lecce, Italy; 3 Istituto per la Protezione Sostenibile delle Piante, CNR – IPSP, Consiglio Nazionale delle Ricerche, Via Amendola 165/A, 70126 Bari, Italy; 4 Dipartimento di Scienze della Vita, Università di Modena e Reggio Emilia, Via Campi 103, 41125 Modena, Italy; 5 Istituto di Nanotecnologia − CNR NANOTEC, Consiglio Nazionale delle Ricerche, Via Monteroni, 73100 Lecce, Italy; 6 Dipartimento di Scienze e Tecnologie Biologiche ed Ambientali, Università del Salento, Via Monteroni, 73100 Lecce, Italy; 7 UdR INSTM, Università del Salento, Lecce, Italy

**Keywords:** Olive quick decline syndrome, Liquid chromatography, High-resolution mass spectrometry, Metabolomics

## Abstract

Olive quick decline syndrome (OQDS) is a disorder associated with bacterial infections caused by *Xylella fastidiosa* subsp*. pauca* ST53 in olive trees. Metabolic profile changes occurring in infected olive trees are still poorly investigated, but have the potential to unravel reliable biomarkers to be exploited for early diagnosis of infections. In this study, an untargeted metabolomic method using high-performance liquid chromatography coupled to quadrupole-time-of-flight high-resolution mass spectrometry (HPLC-ESI-Q-TOF-MS) was used to detect differences in samples (leaves) from healthy (Ctrl) and infected (Xf) olive trees. Both unsupervised and supervised data analysis clearly differentiated the groups. Different metabolites have been identified as potential specific biomarkers, and their characterization strongly suggests that metabolism of flavonoids and long-chain fatty acids is perturbed in Xf samples. In particular, a decrease in the defence capabilities of the host after *Xf* infection is proposed because of a significant dysregulation of some metabolites belonging to flavonoid family. Moreover, oleic acid is confirmed as a putative diffusible signal factor (DSF). This study provides new insights into the host-pathogen interactions and confirms LC-HRMS-based metabolomics as a powerful approach for disease-associated biomarkers discovery in plants.

**Graphical abstract:**

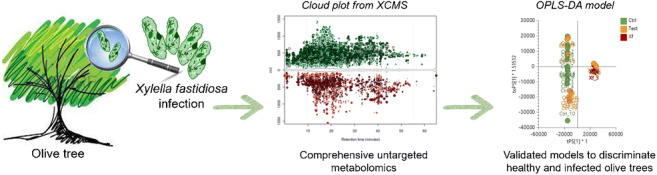

**Supplementary Information:**

The online version contains supplementary material available at 10.1007/s00216-021-03279-7.

## Introduction

Since 2010, olive trees of Salento (southeastern Italy) have been collapsing due to a severe disease called “olive quick decline syndrome” (OQDS) [1]. The disease has been associated with *Xylella fastidiosa* (*Xf*) infection, a Gram-negative pathogen colonizing xylem vessels, thus impairing water uptake. This bacterium is the well-known causal agent of other economically important diseases, i.e. Pierce’s disease [2] in grapes and leaf scorch of almond and other landscape and ornamental species. In the case of olive, the widespread occurrence of sapwood fungal infections (such as *Phaeoacremonium* and *Phaeomoniella*, *Pleumostomophora* and *Neofusicoccum),* probably exacerbates the effect of Xylella-infections [3]. However, the pathogenic role of *X. fastidiosa* subsp. *pauca* ST53 in the aetiology of OQDS has been clearly demonstrated by artificial inoculations on susceptible olive cultivars [4, 5].

This study also unraveled the long incubation period of the infections in olives. In fact, the onset of symptoms occurred more than 1 year after the bacterial inoculation [6]. In this scenario, the possibility of making an early diagnosis of the infections is of utmost importance. To date, it is possible to detect the presence of *Xf* in olive trees by serological and molecular methods [7]. However, the long latency period of the infections and the irregular distribution of the bacterium in the trees (in particular, in century-old trees of relevant size) prompted the exploration of alternative tools to unravel the presence of the infections by detecting bacterial compounds involved in the infection process, including those related to the host response.

Metabolomics emerged within the omics technologies as a valuable aid to understand complex molecular complex in biological systems [8] and to provide a useful evaluation of the cellular state at a molecular level. In fact, metabolites can be observed as the end-products of gene expression and can represent a "fingerprint" of a cell or tissue. Moreover, they generally change within either host or pathogen in response to their specific interactions. In a biological system, the comprehensive analysis of all metabolites is a difficult target, not yet reached for any system. Indeed, the past few years have seen great advances in high-throughput metabolomics, after integration with the other “omics” through bioinformatics [8, 9], making significant progress into understanding different biological processes.

In the recent efforts to comprehend and face OQDS, different analytical procedures have been widely employed to achieve adequate metabolite coverage. A GC-MS method [10] has been adopted to highlight differences in the composition of volatile organic compounds (VOC) between healthy and infected olive tree samples. In a recent work, an LC-MS untargeted lipidomic analysis of infected *Olea europaea* samples revealed a shortlist of molecules that modulate biofilm phases in *X. fastidiosa* subsp. *pauca* [11].

The basic philosophy of an untargeted metabolomics approach is to detect as many metabolites as possible to maximize the likelihood of identifying compounds that are dysregulated in a biological condition. In this research work, using an untargeted metabolomics approach, we attempted to identify metabolites produced by the pathogen or metabolites that represent the biochemical response of the host to the infection. An efficient analytical method based on a liquid chromatography separation coupled to high-resolution mass spectrometry (LC-HRMS) permitted us to unravel the main differences between healthy and *Xylella*-infected trees. Feature extraction and two-group analysis was performed by the XCMS online software. Significant features were used to distinguish samples taken from healthy (Ctrl) and infected (Xf) olive trees and to perform partial least square discriminant analysis. To validate our experimental model, cross-model validation and permutation tests were run. Online databases also allowed for the putative identification of some of the metabolites involved in OQDS. The relevant results are herein discussed.

## Materials and methods

### Sampling

Field samples were collected from olive trees in two different areas of the Salento Peninsula. Ten leaves collected from different brushes of each sample tree were immediately shock-frozen with liquid nitrogen to block all metabolic processes and transferred to the laboratory for extraction. All sampled plants were without OQDS symptoms at different distances from the infected (desiccated) ones. Table S1 (see Supplementary Information, ESM) lists the sample label, the cultivar and healthy/infected state based on real-time PCR assays carried out as already reported [9].

### Extraction protocol and sample preparation

The extraction procedure is a critical step prior to metabolomic experiments. First, the leaves were immersed in liquid nitrogen, then manually ground with a pestle and a mortar (pre-cooled and filled with liquid nitrogen). For the extraction procedure, ethyl acetate was chosen, which was reported to be the best extraction solvent in terms of number of metabolites with large chemical and structural diversity detected by MS [12–14]. Therefore, 300 mg of fine powder was extracted with 1.2 mL of ethyl acetate in 1.5 mL Eppendorf tubes, sonicated for 15 min, and centrifuged at 12,000 rpm for 20 min. 500 μL of the supernatant was transferred into a new Eppendorf tube and the solvent was evaporated under a stream of nitrogen. The addition of 1 mL of water/acetonitrile 50:50 (*v*/v) to the dry extract was followed by sonication (15 min) and centrifugation (12,000 rpm, 20 min). The supernatants were transferred into HPLC vials and analyzed by LC-MS.

### HPLC-ESI-QTOF analysis

Analyses were performed on an Agilent 1200 series liquid chromatograph (LC) equipped with a solvent vacuum degasser, a binary pump, a thermostated autosampler, a thermostated column compartment and a photodiode array detector (DAD) and interfaced to an Agilent 6540 Q-TOF-MS equipped with an ESI source. A general-purpose C18 column (Poroshell 120 SB-C18, 2.1 × 100 mm, 2.7 μm, Agilent, Milano, Italy) was employed and metabolites were separated using a mobile phase composed of 0.1% formic acid in water (A) and 0.1% formic acid in acetonitrile (B). The gradient elution was set as follows: linear gradient from 5 to 95% B in 45 min, 95% B held for 10 min, linear gradient from 95 to 5% B in 5 min and 5% B held for 5 min to equilibrate the column at the initial conditions. The flow rate was 300 μL/min. The run time was 65 min. The column temperature was set at 25 °C. The sample injection volume was 5 μL. DAD acquisitions were carried out in the range of 190–600 nm. The mass spectrometer was operated in full-scan mode in the *m/z* range 50–1700 with a scan rate of 1.42 spectra/s, in both positive and negative ionization modes with the following experimental parameters: the capillary voltage was 3.5 kV, the nebulizer (N_2_) pressure was 30 psi, the drying gas temperature was 350 °C, the drying gas flow was 12 L/min and the skimmer voltage was 40 V. Internal calibration was achieved by enabling constant check of reference masses in the calibration mix solution introduced to the ion source via the dual ESI port. To identify the molecular structure of significant ions, all samples were also acquired in both positive and negative ionization modes using the auto MS/MS function, nitrogen as the collision gas, and a collision energy of 20 eV, under the same experimental conditions described above. All the measurements were run and recorded with Agilent Mass Hunter software (Rev. B.01.04).

### Data processing 

LC/MS data were first processed using XCMS Online software (https://xcmsonline.scripps.edu) [15]. Raw LC-MS data files were converted into *mzML* files and uploaded to the online platform in two datasets, containing 17 Ctrl samples and 10 Xf samples. Additional data from samples of different cultivars (18–22 Ctrl samples) were treated with the same procedure. The following parameters were used to process either positive or negative ion mode data in different pairwise jobs: feature detection with the centWave algorithm (Δ*m/z* = 15 ppm, minimum and maximum peak width 10 and 60 s, respectively; S/N threshold = 6); retention time correction with the usual obiwarp settings (profStep = 0.5); chromatogram alignment with mzwid = 0.015, minfrac = 0.5, and bw = 5; parameters for annotation, including ppm error = 5 and *m/z* absolute error = 0.015. The results of these pairwise calculations are presented in a table in which detailed information about each single metabolite (*m/z*, *p* value, fold-change, peak intensity, retention time, extracted ion chromatogram (EIC), mass spectrum and box plot) are reported. The identification of metabolites was achieved by MS/MS spectrum match with MS-FINDER, which was able to elucidate the formula and chemical structures from accurate mass precursor ions and MS/MS spectra [16]. The XCMS *csv* output file was imported in SIMCA (version 16). This software was used to perform principal component analysis (PCA), partial least square discriminant analysis (PLS-DA) and orthogonal projections to latent structures discriminant analysis (OPLS-DA) and to validate the models. Data were Pareto scaled before performing PCA, PLS-DA and OPLS-DA methods.

## Results

### Data analysis

**Fig. 1:**
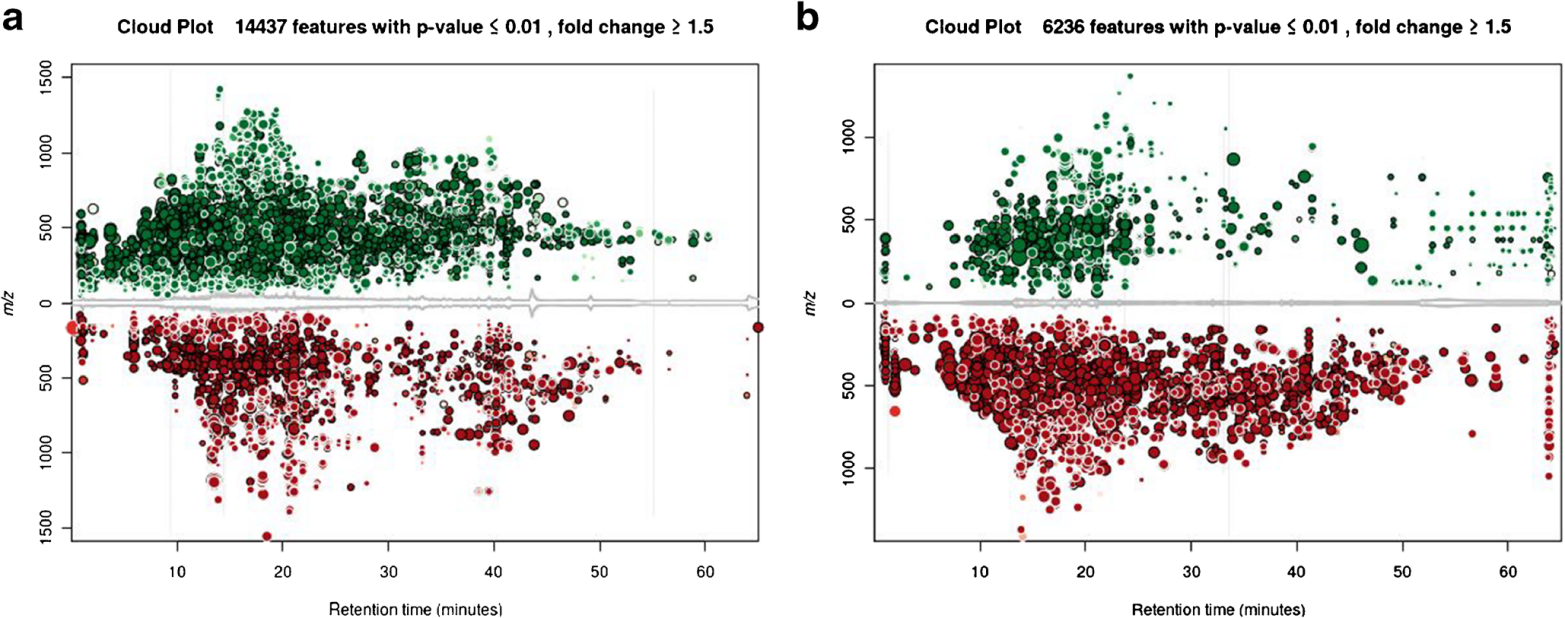
XCMS Cloud plot representation of the dysregulated metabolite features for ESI-MS (**a**) positive ion mode and (**b**) negative ion mode: green bubbles represent the up-regulated features whereas the red ones represent the down-regulated features. Bubble diameter is proportional to fold change

The *mzML* data files were processed with XCMS Online software using pairwise analysis, which allows the comparison of two groups “control” and “disease”, i.e. healthy and Xf-infected plants. The analyses were carried out by ESI-MS in either positive or negative ion mode. The feature detection carried out by XCMS Online with the centWave method permitted us to extract 26,604 and 9598 features for + and – ion mode data, respectively, whereas the parametric independent (unpaired) two-group tests (Welch *t* test) permitted us to determine the metabolite features whose levels are significantly different. Figure 1 gives the actual representation of global metabolomic data in ESI-MS (both + and - ion mode): the relevant cloud plots (that can be exported directly from XCMS Online) show those features exceeding both *p* value and FC thresholds, set at 0.01 and 1.5, respectively, distributed along the chromatogram in the upper or lower panel according being up or downregulated by the Xf infection.

The number of features with a *p* value < 0.01 and fold change > 1.5 were 14.437 in positive ion mode and 6,236 features in negative ion mode. These findings confirm the greatest sensitivity of ESI-MS (+ ion mode) in global metabolomic analysis.

**Fig. 2:**
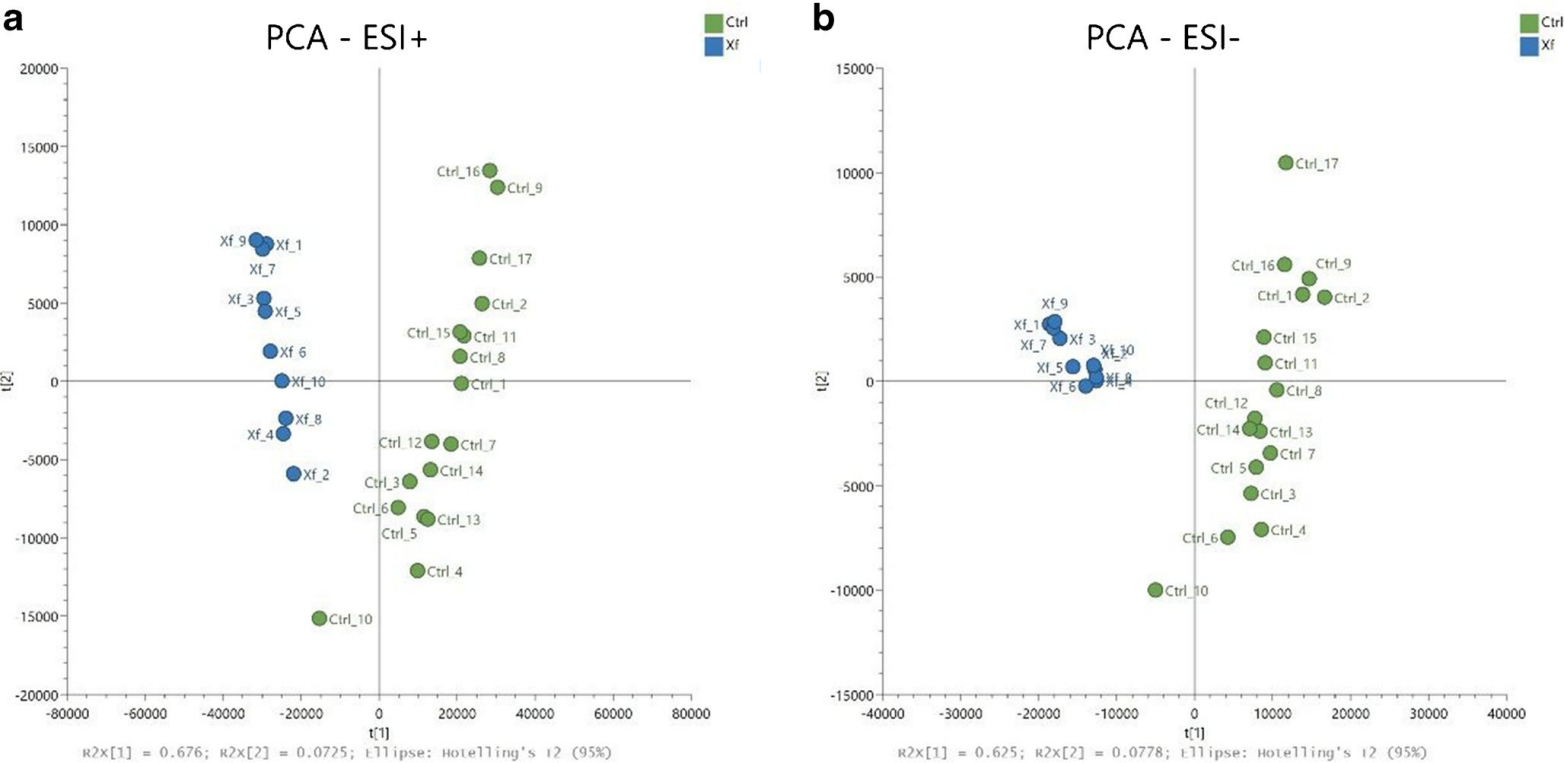
Score plot of principal component analysis (PCA) on metabolomic data acquired in ESI-MS (**a**) positive ion mode and (**b**) negative ion mode

The patterns in the data were searched by PCA, an unsupervised multivariate method useful to examine whether the detected features are able to group samples. This method is “unsupervised” because it is carried out without data labeling with class membership. It is also a strong method to dimensionally reduce a data set containing thousands of metabolites, calculating the few combinations best explaining original data variance. Figure 2 shows the PCA score plots, showing its capability to separate healthy and Xf-infected olive tree samples along PC1 and PC2. The first two PCs explained 67.6% and 7.25% of the total variance in ESI (+ ion mode, panel A) and the 62.5% and 7.7% in ESI (− ion mode, panel B).

These results suggested that the PCA represents an effective tool to distinguish Ctrl (healthy) and Xf (infected) olive trees. Actually, notwithstanding there are two different cultivars, the trend is unique, and no outlier has been identified. Filtering out those features that did not change significantly between the two groups, i.e. using the same features shown in the cloud plot of Fig. 1, the effectiveness of PCA in distinguishing the two groups was confirmed. As a result, a valid classification of these metabolomic data could be obtained [17] and PLS-DA and OPLS-DA were used to further investigate the discrimination ability of these significant metabolites. Both methods are among the most often used supervised method in metabolomics and maximize the covariance between experimental data and dummy-dependent y-variable-containing group labels. Moreover, OPLS-DA permits the separation of group-predictive and group-unrelated variance, calculating the further components orthogonal to the first. The model calibration was performed on about 80% of samples of each class, i.e. on 22 samples, whereas the training set consisted of the remaining ones as reported in Table 1.

**Table 1:** Sample partition in Ctrl (healthy olive trees) and Xf (infected ones) classes, as well as in training and test sets

	Ctrl (healthy olive trees)	Xf (infected olive trees)	Total
Training set	14	8	22
Test set	3	2	5
Total	17	10	27

The selection of latent variables and the cross validation of the model was performed using cancellation groups on the basis of Venetian blinds because the samples are ordered. Obviously, at this point, only the training set (WS or working set in SIMCA) is considered. Two different validation runs (3 and 4 cancellation groups) gave similar results in terms of error rate: in order to not overfit experimental data, 2 latent variables and 3 cancellation groups were selected to calculate the final models. Their performance is summarized in Table 2.

**Table 2:** PLS-DA and OPLS-DA models component description and predictive performances

	Component	R2X	R2X (cum)	Eigenvalue	R2Y	R2Y(cum)	Q2	Q2 (cum)
PLS-DA (ESI-MS data, positive ion mode)	1	0.629	0.629	13.8	0.859	0.859	0.844	0.844
	2	0.0609	0.69	1.34	0.127	0.986	0.464	0.916
PLS-DA (ESI-MS data, negative ion mode)	1	0.601	0.601	13.2	0.83	0.83	0.814	0.814
	2	0.0593	0.66	1.31	0.145	0.975	0.526	0.912
OPLS-DA (ESI-MS data, positive ion mode)	P1 (Predictive)	0.542	0.542	11.9	0.986	0.986	0.917	0.917
	O1 (Orthogonal in X)	0.149	0.149	3.27				
OPLS-DA (ESI-MS data, negative ion mode)	P1 (Predictive)	0.504	0.504	11.1	0.975	0.975	0.874	0.874
	O1 (Orthogonal in X)	0.156	0.156	3.44				

**Fig. 3:**
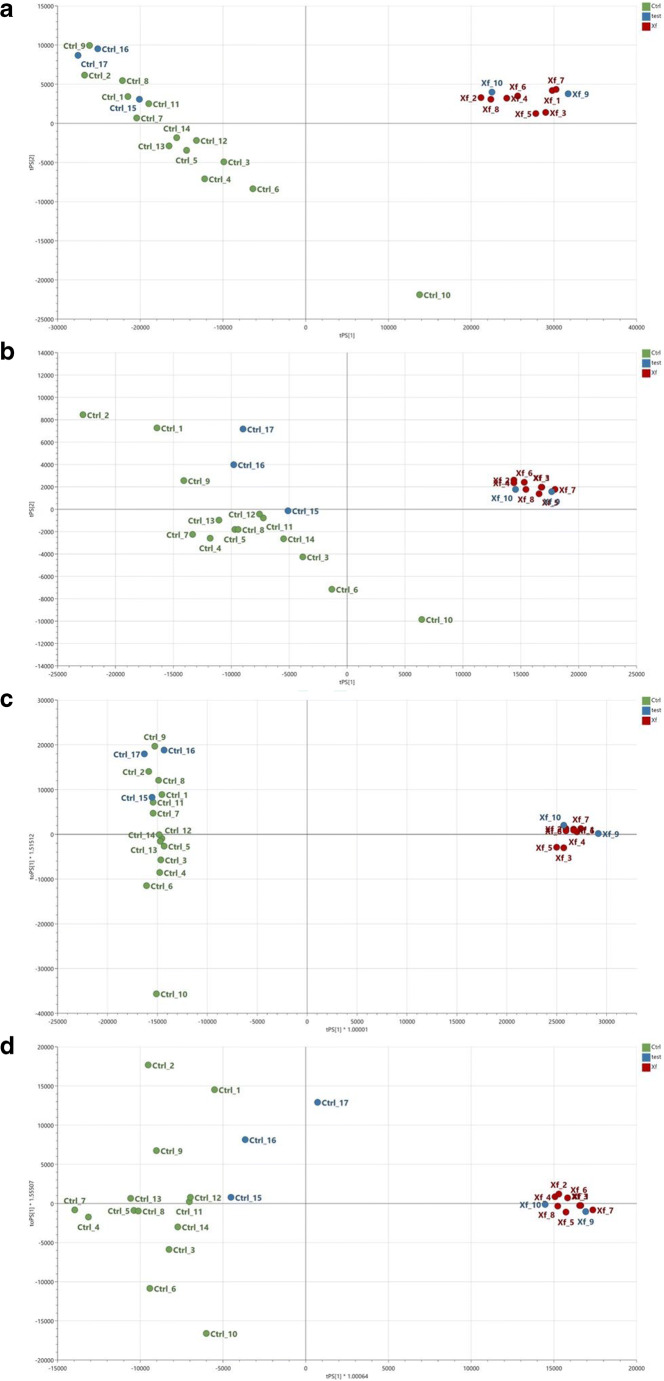
Scatter plots for the predicted scores of the two components retained in the PLS-DA and OPLS-DA models calculated for both ESI-MS ion modes: (**a**) PLS-DA, ESI-MS, + ion mode; (**b**) PLS-DA, ESI-MS, − ion mode; (**c**) OPLS-DA, ESI-MS, + ion mode; and (**d**) OPLS-DA, ESI-MS, − ion mode. Healthy (Ctrl) and Xf-infected samples (Xf) belonging to the training set are coloured in green and in red, respectively, whereas all the test samples are in blue. Variables were filtered according to *p* value <0.01 and fold change > 1.5 for both ESI modes

The reliability of the models can also be ascertained from the obtained PLS-DA (Fig. 3a and b) and OPLS-DA (Fig. 3c and d) score plots: all the samples, in both the training set and the test set, have been plotted in the figures and the modeling capabilities are evident, with a correct classification of all the test samples that have not been used in the model fitting (ESM Table S2). The model is also robust towards samples not belonging to Ogliarola and Cellina, which represent the cultivars that have been considered in the present work: 5 samples of different cultivars, all not affected by OQDS are correctly classified as healthy (see ESM, Fig. S2 and Tables S3-S6).

Feature annotation, i.e. the metabolite identification, is a key step to find those biomolecule markers of the infection that are potentially useful to develop a new analytical method based on a targeted metabolomic approach aiming at an early diagnosis of the infection. Among these thousands of features, only those exhibiting the most significant fold changes were searched in detail. Tentative metabolite identification was based on the in silico MINE database and in the PubChem compound database available in MS-FINDER, which also allows us to score and rank candidate structures. Tables 2 lists some of the significant metabolites with their retention time, accurate m/z value, fragment ions, fold change, *p* value, and ion mode. This software was also used to confirm annotation by comparing the experimental MS/MS spectra with the computer generated ones. The high-resolution MS/MS spectra of metabolites identified and discussed below are reported in Fig. S1 (see ESM).

**Fig. 4:**
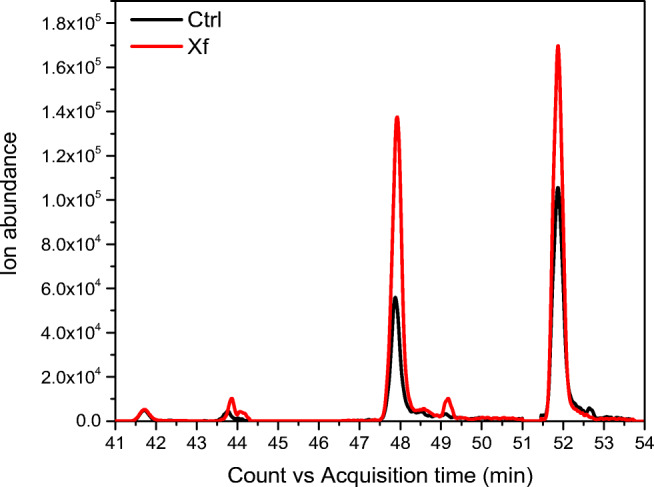
Comparison of extracted ion chromatogram (EIC) of some fatty acid metabolites in ESI-MS (negative ion mode) between Ctrl and Xf-infected samples

As can be seen in Table 3, some flavone and flavonoid-O-glucosides were detected as significantly dysregulated. This result is not surprising because flavonoids are widely distributed and some of them are involved in response to microbial attack in different host-pathogen systems [18]. Changes in the levels of metabolites belonging to the class of fatty acids, both saturated and unsaturated, were also recorded in the chromatograms. In Fig. 4 the extracted ion chromatograms for Ctrl and Xf samples (ESI-MS, − ion mode) shows how palmitic acid and stearic acids are upregulated in Xf-infected samples. Oleic acid was also upregulated, with a 6-fold increase in the Xf samples.

**Table 3:** Significant metabolites identified in healthy (control) and Xf samples whose dysregulation between not-infected and infected plant samples is discussed in the text. For each recognized metabolite, along with electrospray mode, formula, exact mass, and retention time, the values explaining the differences are reported: fold change, *p* value and dysregulation trend. These metabolites have been confirmed by MS/MS experiments (ESM Fig. S1)

Metabolite	ESI-MS ion mode	Formula	*m/z*	RT (min)	Fold change	p value	Change trend
Pyridoxine	+	C_8_H_11_NO_3_	170.0804	1.1	8.4	1.23E-06	UP
β-Ionone	+	C_13_H_20_O	193.1577	11.42	30.2	2.17E-08	UP
Taxifolin	–	C_15_H_12_O_7_	303.0485	12.13	6.7	1.82E-06	DOWN
Kaempferol	–	C_15_H_10_O_6_	285.0376	12.14	5.6	3.69E-06	DOWN
Solavetivone	+	C_15_H_22_O	219.1663	13.19	12.6	4.35E-12	UP
Diosmin	–	C_28_H_32_O_15_	607.1614	13.88	2.0	1.11E-03	DOWN
Diosmetin 7-O-beta-D-glucopyranoside	–	C_22_H_22_O_11_	461.0673	13.88	5.3	6.43E-10	DOWN
Jasmonic acid	–	C_12_H_18_O_3_	209.1180	15.26	8.2	1.22E-07	UP
(S)-Abscisic acid	–	C_15_H_20_O_4_	263.1264	15.67	3.3	9.05E-09	UP
Ligstroside	–	C_25_H_32_O_12_	523.1752	16.09	1.9	3.96E-03	DOWN
Luteolin	–	C_15_H_10_O_6_	285.0376	12.14	5.6	3.69E-07	DOWN
Sinapic acid	+	C_11_H_14_O_5_	225.075	20.35	3.4	3.00E-04	DOWN
Maslinic acid	–	C_30_H_48_O_4_	471.3472	30.50	3.3	2.72E-09	UP
12-HETE	–	C_20_H_32_O_3_	319.2298	32.30	5.0	3.00E-05	DOWN
Palmitic acid	–	C_16_H_32_O_2_	255.2330	36.52	10.5	3.00E-05	UP
Heptadecanoic acid	–	C_17_H_34_O_2_	269.2489	36.86	7.9	4.05E-06	UP
Oleic acid	–	C_18_H_34_O_2_	281.2504	37.76	6.0	5.00E-05	UP
Stearic acid	–	C_18_H_36_O_2_	283.2670	51.87	1.6	6.00E-04	UP

## Discussion

In the present work, we employed an untargeted metabolomics approach to identify the main changes in the metabolic profile of olive leaves affected by the “olive quick decline syndrome”. Significant upregulated or downregulated metabolites detected by ESI-MS (either in + or – ion mode) and reported in Tab. 3 are discussed below.

*Diosmin.* The deprotonated molecule [M-H]^−^ was observed in the ESI-MS (− ion mode) at *m/z* 607.1614. Diosmin is a polyphenol with a flavonoid structure, showing high solubility in water, and its presence appears to be included in defence response activities by blocking protein synthesis in viruses [19]. In our samples, the diosmin was downregulated in Xf samples, which may be ascribed to the defence mechanisms being activated in infected plants.

*Diosmetin 7-O-beta-D-glucopyranoside* has been also identified in ESI-MS (− ion mode) as a downregulated metabolite in *Xf* samples (5.3-fold) at *m/z* 461.0673 and it is often cited as having antioxidant and antimicrobial effects [20].

*Taxifolin* (also known as dihydroquercetin), having a chemical formula of C_15_H_12_O_7_ was identified at *m/z* 303.0485 (ESI-MS, − ion mode). The potential interest in taxifolin is mainly focused on its antioxidant properties. In our results, the amount of taxifolin was downregulated with a 6.7-fold change.

*Ligstroside.* Ligstroside is a glucoside secoiridoid involved in plant metabolism. It is a secondary plant metabolite having a chemical structure containing one phenol ring and a formula of C_25_H_32_O_12_ and was identified at *m/*z 523.1752 in ESI-MS (− ion mode). Ligstroside is generally present in olive trees' lipidic fraction and in extra-virgin olive oil (EVOO), and its antioxidant, anticarcinogenic, anti-inflammatory and immunomodulatory properties have been assessed [21]. In our study, this metabolite is downregulated, and it is discriminative for the infected leaves (1.9-fold change) in comparison with infected ones (Xf).

*Kaempferol and Luteolin.* These metabolites are significantly downregulated, with up to a 5.6-fold change measured for kaempferol. Both kaempferol and luteolin have the same molecular formula and mass and were distinguished by their different MS/MS spectra (ESM Fig. S1). It has been widely reported that flavonoid content in plants is responsible for antimicrobial activity, especially towards Gram-negative bacteria [22].The results in the present study thus illuminate one of the possible effects of microbial attack, i.e. the demolition of the antioxidant potential of flavonoids, paving the way for rapid infection of the Xf-attacked plants.

*Solavetivone* has been identified at *m/z* 219.1653 in ESI-MS (+ ion mode). This is a stress compound with a sesquiterpenoid structure, and is thus implicated in disease resistance [23]. It is most likely produced by the plant as a result of post-infection stress. Given that it is expressed in major concentrations in Xf leaves (12.6-fold higher with respect to the controls), this molecule could represent a biological marker of infection.

*Sinapic acid.* The metabolite having a m/z 225.0747 ESI-MS (+ ion mode) was identified as sinapic acid, which is constitutively expressed in the leaves of healthy trees. But, its concentration decreases (3.4 times) when the plant is in a state of stress.

*Pyridoxine (Vitamin B6).* Pyridoxine has been identified at *m/z* 170.0804 in ESI-MS (+ ion mode). In the context of self-defence against oxidative stress, we found that pyridoxine, one of the six isoforms of vitamin B6, was expressed with concentrations 8.4 times higher by the infected trees, indicating that the plant had implemented its defences against the infection. Moreover, it has been recently reported that vitamin B6 could play a key role as a signalling molecule to alert the plant of the need for ammonium, a nitrogen source necessary for the biosynthesis of vital compounds, such as amino acids and proteins [24].

*Abscisic acid.* The deprotonated plant hormone abscisic acid [M – H]^−^ was found at *m/z* 263.1264 in ESI-MS (− ion mode). Low-energy CID-MS/MS analysis of this precursor ion was initiated by loss of carbon dioxide to afford the [M-CO_2_]^−^ product ion at *m*/*z* 220.97 [25]. In our study, the abscisic acid was upregulated, with a 3.3-fold change. Concurring with the regulation of stomatal aperture and adaptation to drought, low temperature and salinity, this compound clearly plays a role in the plant response to Xf infection [26].

*Jasmonic acid* at *m/*z 209.1180 was identified in ESI-MS (−ion mode) and it is upregulated in samples from infected plants. Considering that jasmonate regulates plant responses to different stresses such as drought [27], this endogenous rise in JA, as observed in the present work, can be seen as a host response to infection.

*Ionone.* Ionone is a volatile organic compound (VOC) whose concentration is about 30 times higher in infected samples, suggesting its implication in the defence-resistance process. As a VOC, indeed, it functions both as a defence factor and as a signalling molecule, [28] and it could have a key role in a range of interactions between plants (allelopathy) and between plants and non-plant organisms.

*Fatty acids (FAs).* It is noteworthy to mention that oleic acid belongs to a class of unsaturated fatty acids which have been proposed to be involved in Xf *quorum sensing*, a cell-to-cell communication system [5, 13]. These pathogen-derived lipid molecules, known as diffusible signal factors (DSF) [29] play a key role in Xf, as they modulate a wide set of biochemical processes. A complex mechanism of DSF regulation, based on population size, influences the expression of several Xf virulence traits, such as biofilm formation, adhesiveness and motility [30]. Moreover, the higher concentration of oleic acid in Xf samples is consistent with recent findings [11], which report a significant accumulation of this compound in Xf-infected olive samples. In light of this finding, we believe that selected FAs may also be considered as valuable markers of OQDS infection.

To summarize, an untargeted metabolomics approach was applied to olive leaf samples with the aim of understanding the main differences between healthy plants and plants with OQDS. An extraction procedure with ethyl acetate was confirmed to be an effective extraction method, permitting the detection of thousands of features. The results of multivariate analysis showed a perfect clustering of the two pools of samples (Ctrl and Xf) based on two principal components (PC1 and PC2). The first allowed the separation of healthy from infected samples, and the second permitted distinction among different cultivars. Based on these data, PLS models were calculated and validated. A few molecules associated with features displaying significant changes between the two data sets could be identified: some are substances implicated in host response to infections, others are involved in Xf *quorum sensing*. A significant dysregulation of some metabolites belonging to the flavonoid family, evidencing a decrease in the host's defence capabilities after *Xf* infection, has been shown for the first time. Once these results are confirmed on a wider sample, they also may be considered as markers for an early diagnosis of OQDS. This would help in planning a strategy for an efficient therapy.

## Supplementary Information

ESM 1 (DOCX 444 kb)

## Data Availability

After acquisition, Agilent MassHunter data sets were converted into .mzML in centroid mode using MSConvert. *mzMLdata* files were uploaded to https://xcmsonline.scripps.edu. The datasets presented in this study can be found in online repositories. All results of the study are accessible in XCMS website with the following name and Job ID: name: **final job ESI(+)MS1; JobID: 1424775** and **name: final job ESI(−)MS1; JobID: 1424728.**
