# Supplementary material for: HPLC-MS/MS method applied to an untargeted metabolomics approach for the diagnosis of “olive quick decline syndrome”
Source: Anal Bioanal Chem. 2021 Mar 25;414(1):465–73. doi: 10.1007/s00216-021-03279-7 (PMC8748322; doi:10.1007/s00216-021-03279-7)
Supplement: Supplementary file 1 — (DOCX 444 kb) [file 216_2021_3279_MOESM1_ESM.docx]

# Supplementary Information

**HPLC-MS/MS method applied to an untargeted metabolomics approach for the diagnosis of “Olive Quick Decline Syndrome”**

**Sabrina Di Masi**^1^**, Giuseppe E. De Benedetto**^2,^ **, Cosimino Malitesta**^1^*, **Maria Saponari**^3^**, Cinzia Citti**^4,5^**, Giuseppe Cannazza**^4,5^**, Giuseppe Ciccarella**^5,6,^*

^1^Laboratorio di Chimica Analitica, Dipartimento di Scienze e Tecnologie Biologiche ed Ambientali, Università del Salento, Via Monteroni, 73100 Lecce, Italy

^2^Laboratorio di Spettrometria di Massa Analitica e Isotopica, Dipartimento di Beni Culturali, Università del Salento, Via Monteroni, 73100 Lecce, Italy

^3^Istituto per la Protezione Sostenibile delle Piante, CNR – IPSP, Consiglio Nazionale delle Ricerche, Via Amendola 165/A, 70126 Bari, Italy

^4^Dipartimento di Scienze della Vita, Università di Modena e Reggio Emilia, Via Campi 103, 41125 Modena, Italy

^5^Istituto di Nanotecnologia − CNR NANOTEC, Consiglio Nazionale delle Ricerche, Via Monteroni, 73100 Lecce, Italy

^6^Dipartimento di Scienze e Tecnologie Biologiche ed Ambientali Università del Salento, Via Monteroni, 73100 Lecce, Italy & UdR INSTM Salento,

*Corresponding authors: [cosimino.malitesta@unisalento.it](mailto:cosimino.malitesta@unisalento.it), [giuseppe.ciccarella@unisalento.it](mailto:giuseppe.ciccarella@unisalento.it)

**Contents of supporting information**

| **No.** | **Contents** | **Pages** |
| --- | --- | --- |
| Figure S1 | High resolution MS/MS spectrum for significant metabolites identified by comparison of experimental MS/MS spectra and in-silico MS/MS spectra computer generated by MS-FINDER. | **2** |
| Figure S2 | OPLS-DA predicted score plots obtained for (a) ESI/MS (+ ion mode) and (b) ESI/MS (– ion mode). | **1** |
| Figure S3 | OPLS-DA Classification lists obtained for (a) ESI/MS (+ ion mode) and (b) ESI/MS (– ion mode). | **1** |
| Table S1 | Sample classification against different cultivars and geolocalised information | **1** |
| Table S2 | Additional samples classification | **1** |
| Table S3 | PLS-DA Classification lists and confusion matrix obtained for ESI-MS data, positive ion mode on all samples belonging to training (Ctrl 1-14 and Xf 1-8), test (Ctrl 15-17 and Xf 9-10) and new (Ctrl 18-22) sets. | **1** |
| Table S4 | PLS-DA Classification lists and confusion matrix obtained for ESI-MS data, negative ion mode on all samples belonging to training (Ctrl 1-14 and Xf 1-8), test (Ctrl 15-17 and Xf 9-10) and new (Ctrl 18-22) sets. | **1** |
| Table S5 | OPLS-DA Classification lists and confusion matrix obtained for ESI-MS data, positive ion mode on all samples belonging to training (Ctrl 1-14 and Xf 1-8), test (Ctrl 15-17 and Xf 9-10) and new (Ctrl 18-22) sets. | **1** |
| Table S6 | OPLS-DA Classification lists and confusion matrix obtained for ESI-MS data, negative ion mode on all samples belonging to training (Ctrl 1-14 and Xf 1-8), test (Ctrl 15-17 and Xf 9-10) and new (Ctrl 18-22) sets. | **1** |

| 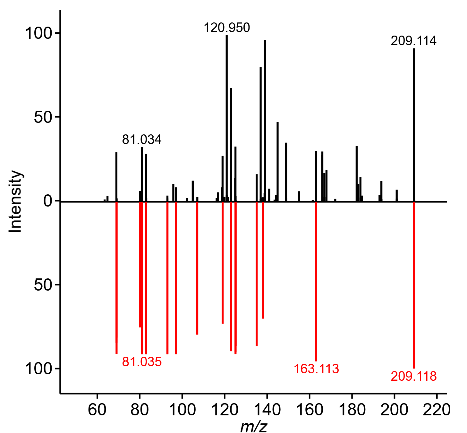 | 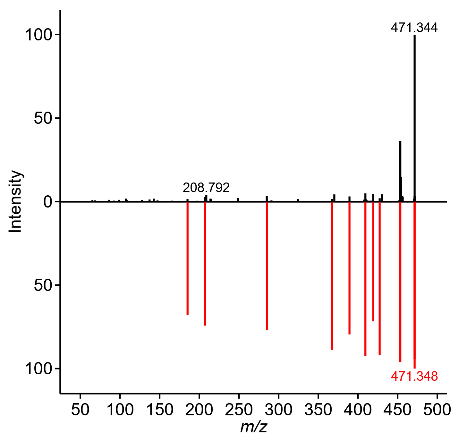 |
| --- | --- |
| **Jasmonic acid**  *m/z:* 209.1180 | **Maslinic acid**  *m/z:* 471.3472 |

| **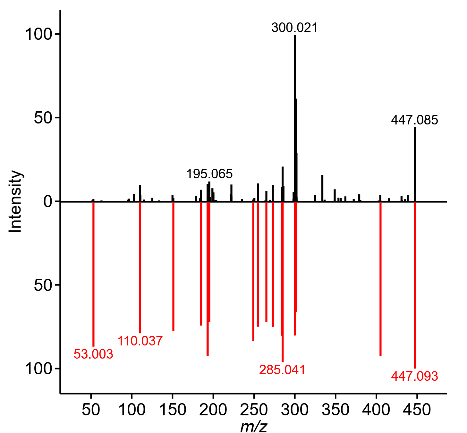** |  | 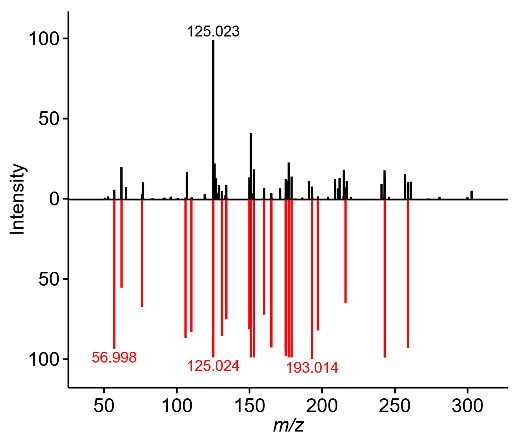 |
| --- | --- | --- |
| **Diosmin**  *m/z:* 607.1614 |  | **Taxifolin**  *m/z:* 303.0485 |

| 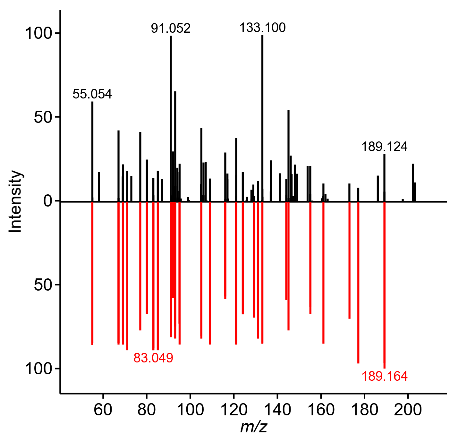 | 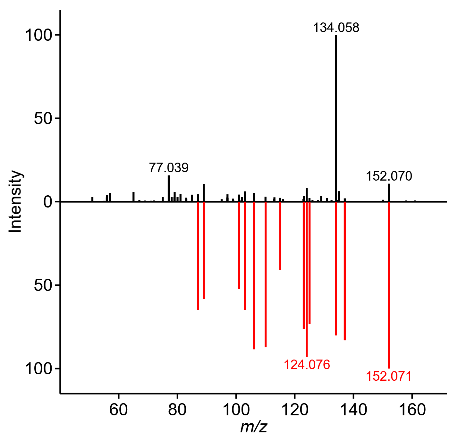 | 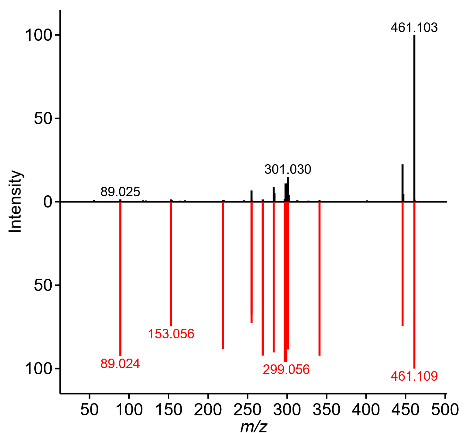 |
| --- | --- | --- |
| **Solavetivone**  *m/z:* 219.1663 | **Pyridoxine (Vitamin B6)**  *m/z:* 170.0804 | **Diosmetin 7-O-beta-D-glucopyranoside**  *m/z*: 461.0673 |

| 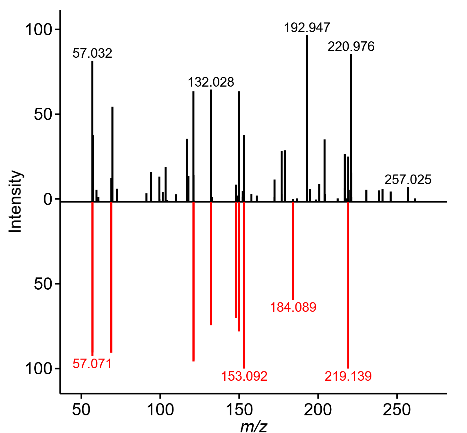 | 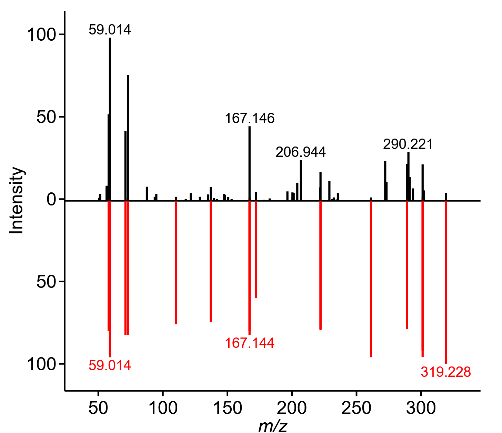 |
| --- | --- |
| **(S)-Abscisic acid**  *m/z:* 263.1264 | **12-HETE**  *m/z:* 319.2298 |

| 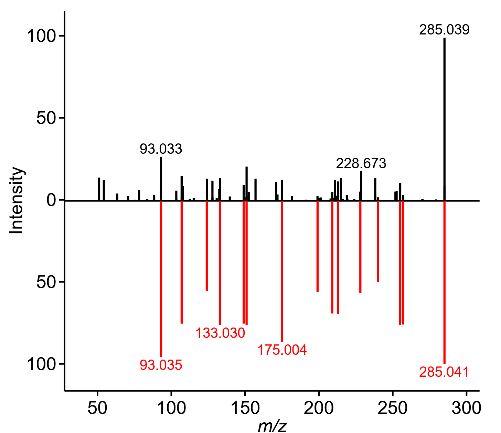 | 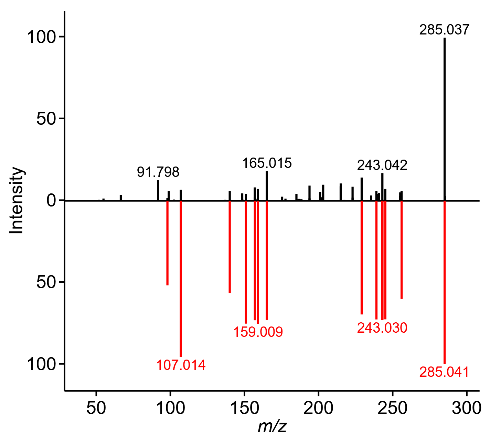 | 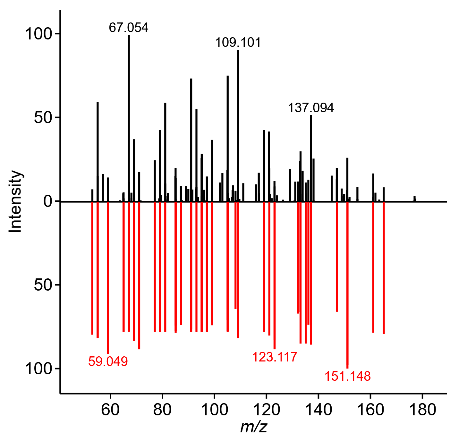 |
| --- | --- | --- |
| **Kaempferol**  *m/z:* 285.0376 | **Luteolin**  *m/z:* 285.0376 | **β-Ionone**  *m/z*: 193.1577 |

Figure S1. High resolution MS/MS spectrum for significant metabolites identified by comparison of experimental MS/MS spectra and in-silico MS/MS spectra computer generated by MS-FINDER.

Table S1. Sample classification

| **Sample no.^1^** | **Cultivar** | **Label** |
| --- | --- | --- |
| 1 | Ogliarola | Ctrl1 |
| 2 | Ogliarola | Ctrl2 |
| 3 | Cellina | Ctrl3 |
| 4 | Cellina | Ctrl4 |
| 5 | Cellina | Ctrl5 |
| 6 | Cellina | Ctrl6 |
| 7 | Ogliarola | Ctrl7 |
| 8 | Ogliarola | Ctrl8 |
| 9 | Ogliarola | Ctrl9 |
| 10 | Cellina | Ctrl10 |
| 11 | Cellina | Ctrl11 |
| 12 | Cellina | Ctrl12 |
| 13 | Cellina | Ctrl13 |
| 14 | Cellina | Ctrl14 |
| 15 | Ogliarola | Ctrl15 |
| 16 | Ogliarola | Ctrl16 |
| 17 | Ogliarola | Ctrl17 |
| 18 | Ogliarola | Xf1 |
| 19 | Cellina | Xf2 |
| 20 | Ogliarola | Xf3 |
| 21 | Cellina | Xf4 |
| 22 | Ogliarola | Xf5 |
| 23 | Ogliarola | Xf6 |
| 24 | Ogliarola | Xf7 |
| 25 | Cellina | Xf8 |
| 26 | Ogliarola | Xf9 |
| 27 | Cellina | Xf10 |

^1^ Samples **1-27** were collected from “Tremolizzo” field

Geolocation: 40°12'56"N 18°20'42"E.

Samples **18-27** were collected from “Uccio” field

Geolocation: 40°01'13"N 18°04'14"E.

Table S2. Additional samples classification

| **Sample no^1^** | **Cultivar** | **Label** |
| --- | --- | --- |
| 28 | Barone di Monteprofico | Ctrl18 |
| 29 | Ornella | Ctrl19 |
| 30 | Usciana | Ctrl20 |
| 31 | Cornola | Ctrl21 |
| 32 | Colozzese | Ctrl22 |

^1^ Samples **28-32** were collected from “Tremolizzo” field

Geolocation: 40°12'56"N 18°20'42"E.

| **a** | **b** |
| --- | --- |
| 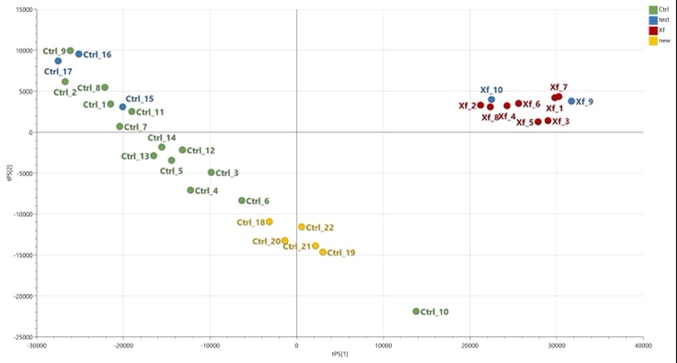 | 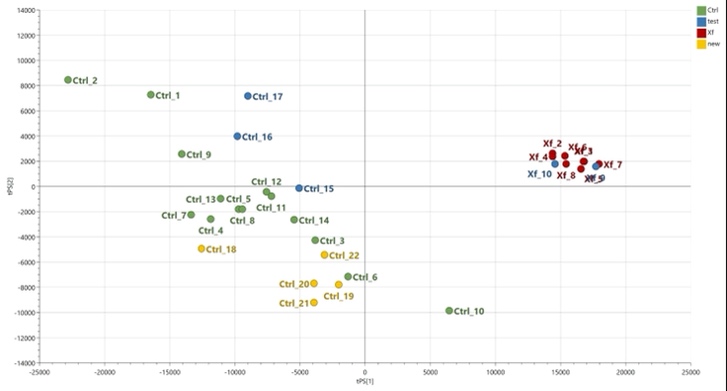 |
| **c** | **d** |
| 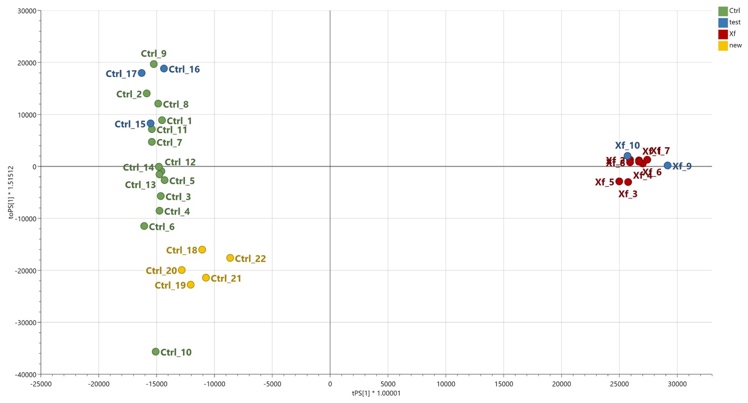 | 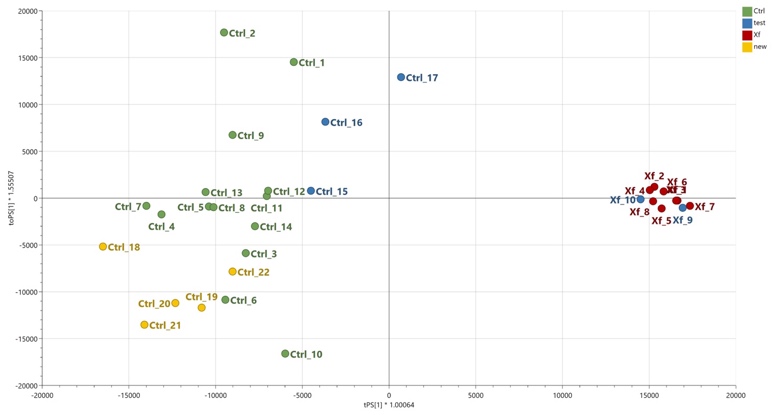 |

Figure S2. Scatter plots for the predicted scores of the two components retained in the PLS-DA and OPLS-DA models calculated for both ESI-MS ion modes: (a) PLS-DA, ESI-MS, + ion mode; (b) PLS-DA, ESI-MS, - ion mode; (c) OPLS-DA, ESI-MS, + ion mode; and (d) OPLS-DA, ESI-MS, - ion mode. Healthy (Ctrl) and Xf infected samples (Xf) belonging to the training set are coloured in green and in red, respectively, whereas all the test samples are in blue. The new samples listes in table S2 are in yellow. Variables were filtered according to p-value <0.01 and fold change >1.5 for both ESI modes.

Table S3. PLS-DA Classification lists and confusion matrix obtained for ESI-MS data, positive ion mode on all samples belonging to training (Ctrl 1-14 and Xf 1-8), test (Ctrl 15-17 and Xf 9-10) and new (Ctrl 18-22) sets.

| **Primary ID** | **$ClassID** | **M1.YVarPS($M1.DA(Ctrl))** | **M1.YPredPS[2]($M1.DA(Ctrl))** | **M1.YVarPS($M1.DA(Xf))** | **M1.YPredPS[2]($M1.DA(Xf))** |
| --- | --- | --- | --- | --- | --- |
| **Ctrl_1** | Ctrl | 1 | 0.992078 | 0 | 0.00792176 |
| **Ctrl_2** | Ctrl | 1 | 1.02918 | 0 | -0.0291808 |
| **Ctrl_3** | Ctrl | 1 | 0.97235 | 0 | 0.0276497 |
| **Ctrl_4** | Ctrl | 1 | 1.07739 | 0 | -0.0773942 |
| **Ctrl_5** | Ctrl | 1 | 1.02722 | 0 | -0.0272187 |
| **Ctrl_6** | Ctrl | 1 | 0.988976 | 0 | 0.0110238 |
| **Ctrl_7** | Ctrl | 1 | 1.04172 | 0 | -0.04172 |
| **Ctrl_8** | Ctrl | 1 | 0.953016 | 0 | 0.0469845 |
| **Ctrl_9** | Ctrl | 1 | 0.916862 | 0 | 0.083138 |
| **Ctrl_10** | Ctrl | 1 | 0.930223 | 0 | 0.0697767 |
| **Ctrl_11** | Ctrl | 1 | 0.964792 | 0 | 0.0352083 |
| **Ctrl_12** | Ctrl | 1 | 0.967998 | 0 | 0.0320025 |
| **Ctrl_13** | Ctrl | 1 | 1.05616 | 0 | -0.0561625 |
| **Ctrl_14** | Ctrl | 1 | 1.00932 | 0 | -0.00932 |
| **Ctrl_15** | test | 0 | 0.971351 | 0 | 0.0286492 |
| **Ctrl_16** | test | 0 | 0.905124 | 0 | 0.0948758 |
| **Ctrl_17** | test | 0 | 0.979076 | 0 | 0.0209236 |
| **Xf_1** | Xf | 0 | -0.0938312 | 1 | 1.09383 |
| **Xf_2** | Xf | 0 | 0.107478 | 1 | 0.892522 |
| **Xf_3** | Xf | 0 | -0.0028577 | 1 | 1.00286 |
| **Xf_4** | Xf | 0 | 0.0467572 | 1 | 0.953243 |
| **Xf_5** | Xf | 0 | 0.0237636 | 1 | 0.976236 |
| **Xf_6** | Xf | 0 | 0.0102778 | 1 | 0.989722 |
| **Xf_7** | Xf | 0 | -0.108296 | 1 | 1.1083 |
| **Xf_8** | Xf | 0 | 0.0894172 | 1 | 0.910583 |
| **Xf_9** | test | 0 | -0.123369 | 0 | 1.12337 |
| **Xf_10** | test | 0 | 0.06371 | 0 | 0.93629 |
| **Ctrl_18** | new | 0 | 0.992576 | 0 | 0.00742432 |
| **Ctrl_19** | new | 0 | 0.962259 | 0 | 0.0377412 |
| **Ctrl_20** | new | 0 | 1.01631 | 0 | -0.0163111 |
| **Ctrl_21** | new | 0 | 0.958631 | 0 | 0.0413686 |
| **Ctrl_22** | new | 0 | 0.932155 | 0 | 0.0678448 |
|  |  |  |  |  |  |
|  | **Members** | **Correct** | **Ctrl** | **Xf** | **No class (YPred <= 0)** |
| **Ctrl** | 14 | 100% | 14 | 0 | 0 |
| **Xf** | 8 | 100% | 0 | 8 | 0 |
| **No class** | 10 |  | 8 | 2 | 0 |
| Total | 32 | 100% | 22 | 10 | 0 |

Table S4. PLS-DA Classification lists and confusion matrix obtained for ESI-MS data, negative ion mode on all samples belonging to training (Ctrl 1-14 and Xf 1-8), test (Ctrl 15-17 and Xf 9-10) and new (Ctrl 18-22) sets.

| **Primary ID** | **$ClassID** | **M2.YVarPS($M2.DA(Ctrl))** | **M2.YPredPS[2]($M2.DA(Ctrl))** | **M2.YVarPS($M2.DA(Xf))** | **M2.YPredPS[2]($M2.DA(Xf))** |
| --- | --- | --- | --- | --- | --- |
| **Ctrl_1** | Ctrl | 1 | 0.85052 | 0 | 0.14948 |
| **Ctrl_2** | Ctrl | 1 | 1.00641 | 0 | -0.0064102 |
| **Ctrl_3** | Ctrl | 1 | 0.9578 | 0 | 0.0421996 |
| **Ctrl_4** | Ctrl | 1 | 1.14632 | 0 | -0.146322 |
| **Ctrl_5** | Ctrl | 1 | 1.03985 | 0 | -0.0398515 |
| **Ctrl_6** | Ctrl | 1 | 1.00307 | 0 | -0.0030694 |
| **Ctrl_7** | Ctrl | 1 | 1.17939 | 0 | -0.179388 |
| **Ctrl_8** | Ctrl | 1 | 1.02984 | 0 | -0.0298429 |
| **Ctrl_9** | Ctrl | 1 | 0.986542 | 0 | 0.0134578 |
| **Ctrl_10** | Ctrl | 1 | 0.869316 | 0 | 0.130684 |
| **Ctrl_11** | Ctrl | 1 | 0.910215 | 0 | 0.0897855 |
| **Ctrl_12** | Ctrl | 1 | 0.90703 | 0 | 0.0929702 |
| **Ctrl_13** | Ctrl | 1 | 1.04787 | 0 | -0.0478666 |
| **Ctrl_14** | Ctrl | 1 | 0.936176 | 0 | 0.0638244 |
| **Ctrl_15** | test | 0 | 0.811275 | 0 | 0.188725 |
| **Ctrl_16** | test | 0 | 0.778613 | 0 | 0.221387 |
| **Ctrl_17** | test | 0 | 0.609077 | 0 | 0.390923 |
| **Xf_1** | Xf | 0 | -0.0102178 | 1 | 1.01022 |
| **Xf_2** | Xf | 0 | 0.0416574 | 1 | 0.958343 |
| **Xf_3** | Xf | 0 | -0.0069686 | 1 | 1.00697 |
| **Xf_4** | Xf | 0 | 0.0514091 | 1 | 0.948591 |
| **Xf_5** | Xf | 0 | 0.0257626 | 1 | 0.974237 |
| **Xf_6** | Xf | 0 | 0.020685 | 1 | 0.979315 |
| **Xf_7** | Xf | 0 | -0.0377318 | 1 | 1.03773 |
| **Xf_8** | Xf | 0 | 0.0450537 | 1 | 0.954946 |
| **Xf_9** | test | 0 | -0.0210642 | 0 | 1.02106 |
| **Xf_10** | test | 0 | 0.0734985 | 0 | 0.926501 |
| **Ctrl_18** | new | 0 | 1.27708 | 0 | -0.27708 |
| **Ctrl_19** | new | 0 | 1.05601 | 0 | -0.0560133 |
| **Ctrl_20** | new | 0 | 1.11515 | 0 | -0.115153 |
| **Ctrl_21** | new | 0 | 1.18487 | 0 | -0.184868 |
| **Ctrl_22** | new | 0 | 0.986662 | 0 | 0.0133381 |
|  |  |  |  |  |  |
|  | **Members** | **Correct** | **Ctrl** | **Xf** | **No class (YPred <= 0)** |
| **Ctrl** | 14 | 100% | 14 | 0 | 0 |
| **Xf** | 8 | 100% | 0 | 8 | 0 |
| **No class** | 10 |  | 8 | 2 | 0 |
| Total | 32 | 100% | 22 | 10 | 0 |

Table S5. OPLS-DA Classification lists and confusion matrix obtained for ESI-MS data, positive ion mode on all samples belonging to training (Ctrl 1-14 and Xf 1-8), test (Ctrl 15-17 and Xf 9-10) and new (Ctrl 18-22) sets.

| **Primary ID** | **$ClassID** | **M2.YVarPS($M2.DA(Ctrl))** | **M2.YPredPS[1]($M2.DA(Ctrl))** | **M2.YVarPS($M2.DA(Xf))** | **M2.YPredPS[1]($M2.DA(Xf))** |
| --- | --- | --- | --- | --- | --- |
| **Ctrl_1** | Ctrl | 1 | 0.992078 | 0 | 0.0079217 |
| **Ctrl_2** | Ctrl | 1 | 1.02918 | 0 | -0.0291808 |
| **Ctrl_3** | Ctrl | 1 | 0.97235 | 0 | 0.0276496 |
| **Ctrl_4** | Ctrl | 1 | 1.07739 | 0 | -0.0773942 |
| **Ctrl_5** | Ctrl | 1 | 1.02722 | 0 | -0.0272188 |
| **Ctrl_6** | Ctrl | 1 | 0.988976 | 0 | 0.0110238 |
| **Ctrl_7** | Ctrl | 1 | 1.04172 | 0 | -0.0417201 |
| **Ctrl_8** | Ctrl | 1 | 0.953016 | 0 | 0.0469844 |
| **Ctrl_9** | Ctrl | 1 | 0.916862 | 0 | 0.083138 |
| **Ctrl_10** | Ctrl | 1 | 0.930223 | 0 | 0.0697768 |
| **Ctrl_11** | Ctrl | 1 | 0.964792 | 0 | 0.0352083 |
| **Ctrl_12** | Ctrl | 1 | 0.967998 | 0 | 0.0320025 |
| **Ctrl_13** | Ctrl | 1 | 1.05616 | 0 | -0.0561625 |
| **Ctrl_14** | Ctrl | 1 | 1.00932 | 0 | -0.0093201 |
| **Ctrl_15** | test | 0 | 0.971351 | 0 | 0.0286492 |
| **Ctrl_16** | test | 0 | 0.905124 | 0 | 0.0948758 |
| **Ctrl_17** | test | 0 | 0.979077 | 0 | 0.0209235 |
| **Xf_1** | Xf | 0 | -0.0938313 | 1 | 1.09383 |
| **Xf_2** | Xf | 0 | 0.107478 | 1 | 0.892522 |
| **Xf_3** | Xf | 0 | -0.0028578 | 1 | 1.00286 |
| **Xf_4** | Xf | 0 | 0.046757 | 1 | 0.953243 |
| **Xf_5** | Xf | 0 | 0.0237635 | 1 | 0.976237 |
| **Xf_6** | Xf | 0 | 0.0102777 | 1 | 0.989722 |
| **Xf_7** | Xf | 0 | -0.108296 | 1 | 1.1083 |
| **Xf_8** | Xf | 0 | 0.0894172 | 1 | 0.910583 |
| **Xf_9** | test | 0 | -0.12337 | 0 | 1.12337 |
| **Xf_10** | test | 0 | 0.06371 | 0 | 0.93629 |
| **Ctrl_18** | new | 0 | 0.992576 | 0 | 0.00742435 |
| **Ctrl_19** | new | 0 | 0.962259 | 0 | 0.0377412 |
| **Ctrl_20** | new | 0 | 1.01631 | 0 | -0.0163112 |
| **Ctrl_21** | new | 0 | 0.958631 | 0 | 0.0413686 |
| **Ctrl_22** | new | 0 | 0.932155 | 0 | 0.0678448 |
|  |  |  |  |  |  |
|  | **Members** | **Correct** | **Ctrl** | **Xf** | **No class (YPred <= 0)** |
| **Ctrl** | 14 | 100% | 14 | 0 | 0 |
| **Xf** | 8 | 100% | 0 | 8 | 0 |
| **No class** | 10 |  | 8 | 2 | 0 |
| Total | 32 | 100% | 22 | 10 | 0 |

Table S6. OPLS-DA Classification lists and confusion matrix obtained for ESI-MS data, negative ion mode on all samples belonging to training (Ctrl 1-14 and Xf 1-8), test (Ctrl 15-17 and Xf 9-10) and new (Ctrl 18-22) sets.

| **Primary ID** | **$ClassID** | **M1.YVarPS($M1.DA(Ctrl))** | **M1.YPredPS[1]($M1.DA(Ctrl))** | **M1.YVarPS($M1.DA(Xf))** | **M1.YPredPS[1]($M1.DA(Xf))** |
| --- | --- | --- | --- | --- | --- |
| **Ctrl_1** | Ctrl | 1 | 0.85052 | 0 | 0.14948 |
| **Ctrl_2** | Ctrl | 1 | 1.00641 | 0 | -0.0064102 |
| **Ctrl_3** | Ctrl | 1 | 0.9578 | 0 | 0.0421996 |
| **Ctrl_4** | Ctrl | 1 | 1.14632 | 0 | -0.146322 |
| **Ctrl_5** | Ctrl | 1 | 1.03985 | 0 | -0.0398514 |
| **Ctrl_6** | Ctrl | 1 | 1.00307 | 0 | -0.0030694 |
| **Ctrl_7** | Ctrl | 1 | 1.17939 | 0 | -0.179388 |
| **Ctrl_8** | Ctrl | 1 | 1.02984 | 0 | -0.0298429 |
| **Ctrl_9** | Ctrl | 1 | 0.986542 | 0 | 0.0134578 |
| **Ctrl_10** | Ctrl | 1 | 0.869316 | 0 | 0.130684 |
| **Ctrl_11** | Ctrl | 1 | 0.910215 | 0 | 0.0897855 |
| **Ctrl_12** | Ctrl | 1 | 0.90703 | 0 | 0.0929702 |
| **Ctrl_13** | Ctrl | 1 | 1.04787 | 0 | -0.0478666 |
| **Ctrl_14** | Ctrl | 1 | 0.936176 | 0 | 0.0638244 |
| **Ctrl_15** | test | 0 | 0.811275 | 0 | 0.188725 |
| **Ctrl_16** | test | 0 | 0.778613 | 0 | 0.221387 |
| **Ctrl_17** | test | 0 | 0.609077 | 0 | 0.390923 |
| **Xf_1** | Xf | 0 | -0.0102177 | 1 | 1.01022 |
| **Xf_2** | Xf | 0 | 0.0416575 | 1 | 0.958342 |
| **Xf_3** | Xf | 0 | -0.0069686 | 1 | 1.00697 |
| **Xf_4** | Xf | 0 | 0.0514091 | 1 | 0.948591 |
| **Xf_5** | Xf | 0 | 0.0257626 | 1 | 0.974237 |
| **Xf_6** | Xf | 0 | 0.020685 | 1 | 0.979315 |
| **Xf_7** | Xf | 0 | -0.0377317 | 1 | 1.03773 |
| **Xf_8** | Xf | 0 | 0.0450537 | 1 | 0.954946 |
| **Xf_9** | test | 0 | -0.0210642 | 0 | 1.02106 |
| **Xf_10** | test | 0 | 0.0734985 | 0 | 0.926501 |
| **Ctrl_18** | new | 0 | 1.27708 | 0 | -0.27708 |
| **Ctrl_19** | new | 0 | 1.05601 | 0 | -0.0560133 |
| **Ctrl_20** | new | 0 | 1.11515 | 0 | -0.115153 |
| **Ctrl_21** | new | 0 | 1.18487 | 0 | -0.184868 |
| **Ctrl_22** | new | 0 | 0.986662 | 0 | 0.0133381 |
|  |  |  |  |  |  |
|  | **Members** | **Correct** | **Ctrl** | **Xf** | **No class (YPred <= 0)** |
| **Ctrl** | 14 | 100% | 14 | 0 | 0 |
| **Xf** | 8 | 100% | 0 | 8 | 0 |
| **No class** | 10 |  | 8 | 2 | 0 |
| Total | 32 | 100% | 22 | 10 | 0 |
